# Evidence of Thrombogenesis Recurrence Induced by IgA Antiphospholipid Antibody β2 Glycoprotein I-Dependent in Early Adulthood

**DOI:** 10.7759/cureus.23535

**Published:** 2022-03-27

**Authors:** Mirna S Yacoub, Justin Khine, Ali Najar, Sri Yadlapalli

**Affiliations:** 1 Department of Internal Medicine, Saint Joseph Mercy Oakland Hospital, Pontiac, USA

**Keywords:** thrombosis, iga isotype, b2 glycoprotein-i, antiphospholipid syndrome, antiphospholipid antibodies

## Abstract

Antiphospholipid antibodies (aPLs) against Beta-2 glycoprotein-I (β_2_GPI) are considered to be the center of pathogenesis of antiphospholipid syndrome (APS). Autoimmune aPLs are pathogenic as patients are at increased risk of enhancing thrombin generation at a young age. There are only three aPLs considered as diagnostic laboratory markers for APS - IgM, IgG, and IgA isotypes. However, the association of the IgA isotypes with clinical thrombosis remains highly controversial.

A 30-year-old male with a past medical history of childhood asthma initially presented to the hospital with acute left middle cerebral artery ischemic stroke, which did not get resolved with tissue plasminogen activator (tPA) but was successfully resolved with thromboembolectomy. It was speculated to be associated with a clot from mitral valve prolapse found subsequently on echocardiogram. Twenty-eight days later, the patient presented again with a high-grade luminal narrowing of his mid- and distal left internal carotid artery with 80% narrowing and an acute dissection of his left internal carotid artery. The recurrence of thrombosis was evaluated through hypercoagulable state workup, which demonstrated evidence of antiphospholipid syndrome with elevated beta-2 glycoprotein IgA antibody titers of more than 150 U/mL. This is one of the first cases reported nationwide as evidence of thrombogenesis recurrence induced by IgA antiphospholipid antibody β_2_ glycoprotein I-dependent in early adulthood.

IgA anti- β_2_GPI antibodies are found to have an association with many clinical manifestations of antiphospholipid syndrome and thrombotic events, particularly arterial thrombosis. To determine the link between the IgA-aβ_2_GPI antibodies and APS-events in asymptomatic individuals before recommending preventive treatments, there needs to be a broader intention to standardize IgA-aβ_2_GPI assays as a diagnostic criterion for APS.

## Introduction

Antiphospholipid antibodies, aPLs in short, are originally considered heterogeneous groups of immunoglobulins that bind to anionic phospholipids. In fact, they are best characterized to directly bind against specific phospholipid-binding proteins [1]. They happen to present in the serum of patients with rheumatic diseases, malignancies, and infections, yet they can also appear in healthy individuals. There are three known APLs to date: lupus anticoagulant (LA), anticardiolipin antibodies (aCL), and anti-β2 glycoprotein-I antibodies (aβ2GPI). The most notable protein in recent studies is beta2 (β2) glycoprotein-I [2].

Autoantibodies against β2GPI have been considered to be the central pathogenesis of antiphospholipid syndrome (APS). Studies showed that anti-β2GPI antibodies are associated with both thrombosis and pregnancy loss in individuals with APS [1].

Autoimmune aPLs are pathogenic as patients with aPLs are at increased risk for thrombosis, as well as showing signs of a prothrombotic (hypercoagulable) state with elevated tissue factor (TF) expression and enhanced thrombin generation. The presence of aPLs and at least one clinical feature of either vascular thrombosis or pregnancy morbidity define the systemic autoimmune disorder “antiphospholipid syndrome”. In the consensus established in Sydney, Australia, during the 11th International Congress of aPL in 2004, there are only three aPL considered as diagnostic laboratory markers for APS: IgM, IgG, and IgA isotypes [3].

The 13th International Congress on antiphospholipid antibodies that took place in 2010 recommended IgA-associated anti-beta-2 glycoprotein-I antibodies (IgA-aβ2GPI) as a laboratory criterion of APS in patients with clinical manifestations but negative for “consensus” aPL (IgG and IgM) isotypes. To date, there have been outstanding studies that have evaluated the risk of IgM and IgG antibodies in manifesting thrombogenesis. However, IgA-aβ2GPI antibodies are not included in the diagnostic protocols of APS since they are not included in the consensus criteria. Therefore, the pathogenesis of those antibodies remains unrecognized [4].

## Case presentation

**Figure 1:**
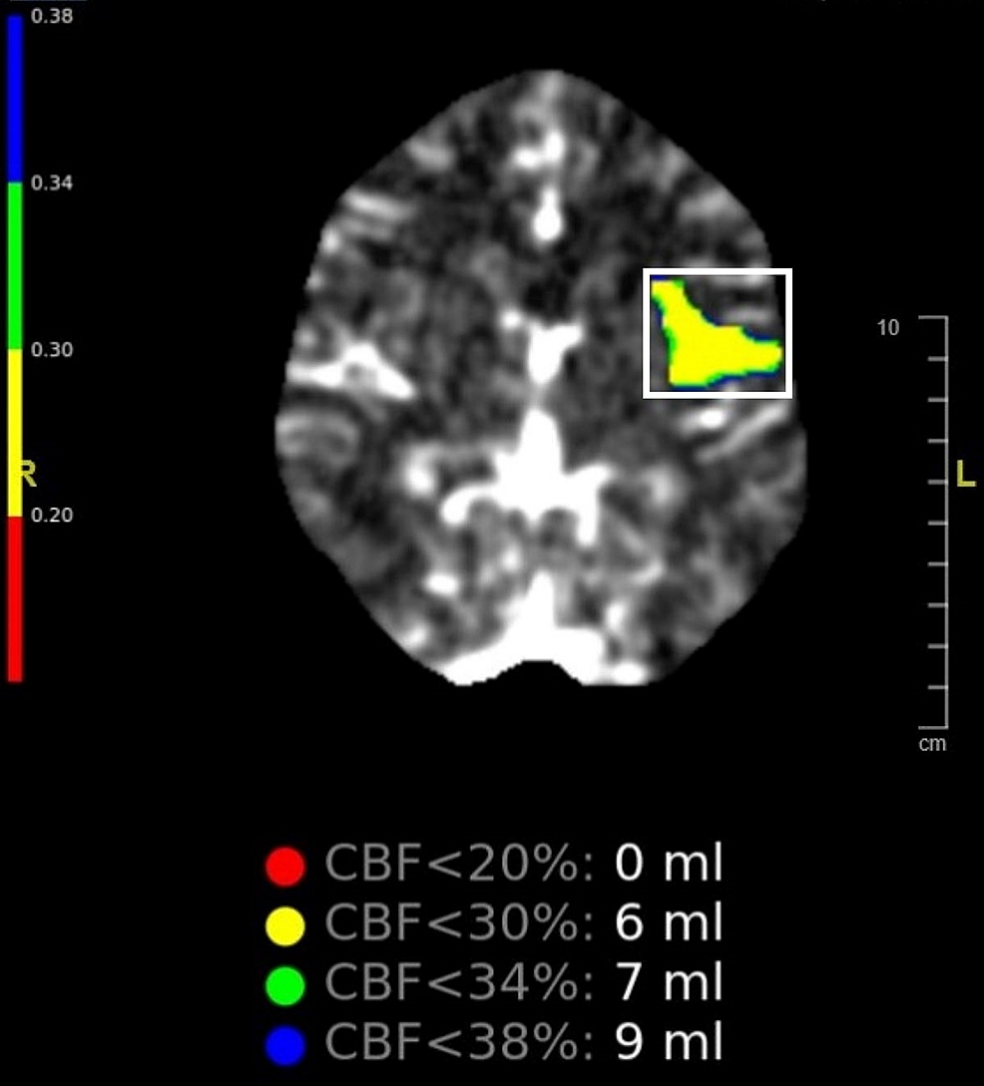
CT Angiography of the brain indicates area of relative perfusion deficits in the left middle cerebral artery (MCA) territory Cerebral blood flow of left MCA territory suggestive of 6mL of core infarct with less than 30% volume, shown in yellow color within a white box.

A 30-year-old male with a past medical history of childhood asthma not currently on medication, mitral valve prolapse with trace mitral regurgitation found incidentally on a transesophageal echocardiogram (TEE), initially presented at the hospital with a sudden loss in the ability to speak and gradually had difficulty moving both of his arms and legs while he was on a bike ride with his family. The patient was immediately taken to the emergency department within four hours of the onset of symptoms. Per the ED physician’s evaluation, the NIH stroke scale/score was six for aphasia and dysarthria. CT head without contrast was done and showed no acute intracranial hemorrhage. CT cerebral perfusion showed acute left middle cerebral artery (MCA) ischemic stroke suggestive of 6 milliliters of core infarct (Figure 1). Within two hours of the onset of stroke symptoms, the patient was given a recombinant tissue plasminogen activator (tPA) intravenously immediately, but he did not respond to the treatment. Intervention with mechanical thromboembolectomy of left MCA occlusion was done after two hours of receiving tPA, which successfully resulted in complete resolution of all of the symptoms.

The patient received the COVID-19 vaccine and denied the use of any medications at home. The patient denied any history of COVID-19 infection, infective endocarditis, transient ischemic attacks, or migraines. Family history was remarkable for stroke in maternal grandmother at age 71, with no history of clotting or bleeding disorders. He denied any use of tobacco smoke, alcohol, or recreational drugs. He has a career as a police officer and maintained a healthy lifestyle with strength exercising of 15 minutes of sit-ups and push-ups three times a week.

Objective findings included a blood pressure of 150/89 mmHg, a pulse of 92 beats per minute, respiratory rate of 22 breaths per minute, and oxygen saturation of 97% on room air. The patient was alert and oriented but showed significant expressive aphasia. There was no Marfanoid features or joint hypermobility noted. The lungs were clear to auscultation bilaterally. Cardiac auscultation revealed regular rate and rhythm, S1 and S2 heart sounds with no extra heart sounds, murmurs, or rubs. The abdomen was soft, non-tender, with bowel sounds present. The patient showed expressive aphasia with no focal neurological deficits were found on the neurological exam; strength of upper and lower extremities was 5/5 bilaterally. His finger-to-nose coordination was intact. Cranial nerves II to XII were intact. Laboratory work-up showed no remarkable findings for complete blood counts (CBC), complete metabolic panel (CMP), prothrombin time (PT) and partial thromboplastin time (PTT), thyroid panel, and vitamin B12 levels. Also, there were no remarkable findings for ongoing inflammation as C-reactive protein (CRP), and erythrocyte sedimentation rate (ESR) were normal.

During the patient’s stay at the hospital, cardiology was consulted, and TEE was performed, which showed a mobile thickened filamentous echo density attached to the mitral valve apparatus unlikely to be vegetation with no left atrial appendage thrombus, patent foramen ovali, or atrial septal defect. There was trace mitral regurgitation with no evidence of mitral stenosis. After seven days of observation at the hospital, the patient was discharged with rivaroxaban in addition to daily aspirin and instructed to follow up with a cardiologist in two weeks. The patient was also continued with atorvastatin and metoprolol. 

**Figure 2:**
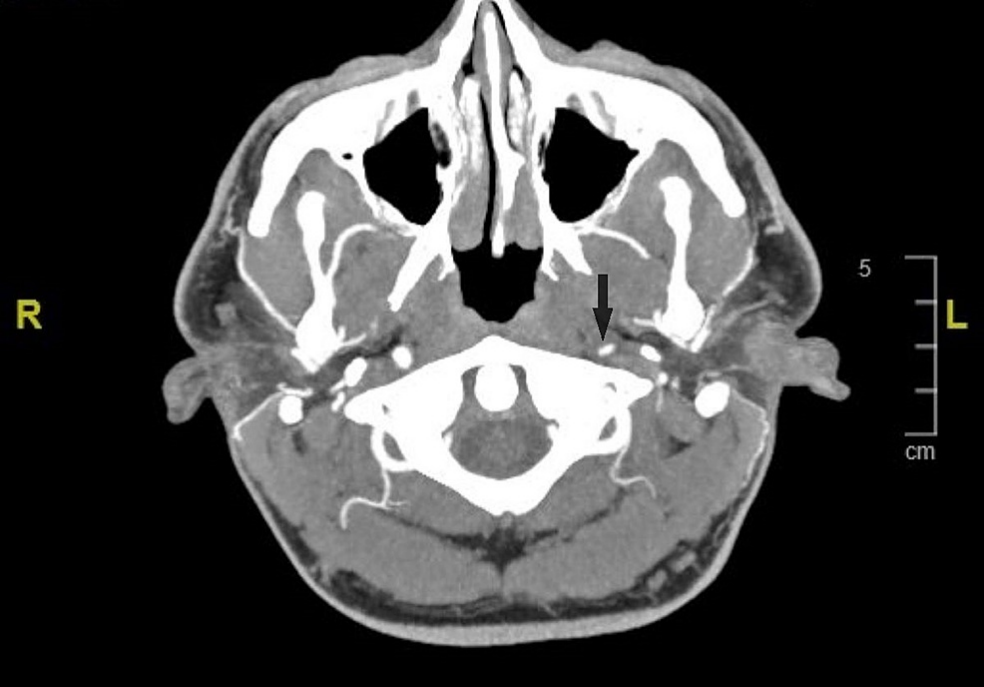
CT angiography of the head shows a high-grade luminal narrowing of mid and distal left internal carotid artery Arrow points to left internal carotid artery with 80% luminal narrowing.

Twenty-eight days later, the patient was again admitted to the hospital after experiencing right lower extremity pain associated with numbness and spontaneous bruising. The patient stated he was sitting on his home’s couch watching TV when he suddenly felt severe pain in his right lower leg and when he looked, he noted bruising. Urinalysis with a reflex microscope showed no remarkable findings. Urine drug screening was also done with unremarkable findings. He had a vascular ultrasound duplex of the right leg which showed no evidence of deep vein thrombosis. He had CT angiography of the head and neck, which showed a high-grade luminal narrowing of 80% of his mid- and distal left internal carotid artery (Figure 2). An MRI of the brain showed no acute stroke but did show an acute dissection of his left internal carotid artery. This was assumed to be secondary to his previous middle cerebral artery thrombectomy. Subsequently, it was treated with stenting, and he was started on ticagrelor, for which he developed pruritus as an allergic reaction, thus, he was continued with rivaroxaban and aspirin.

The patient was referred to the hematologist for hypercoagulable state work-up, which demonstrated evidence of antiphospholipid syndrome with elevated beta-2 glycoprotein IgA antibody titers of more than 150 U/mL (Table 1). Additional anti-coagulation workup showed normal levels of anti-cardiolipin antibodies, protein C activity, and lupus anticoagulants with a negative antinuclear antibody test. There was no evidence of Factor V Leiden mutation. After seven days of hospital stay, the patient was discharged with rivaroxaban and instructed to follow up with a hematologist immediately.

**Table 1:** Beta-2-glycoprotein I antibodies for IgG, IgM, IgA Beta-2-glycoprotein I antibodies with their respective values and reference ranges. Note the comparison of values between IgG, IgM, and IgA

Component	Value	Ref. Range
Beta-2-Glycoprotein I IgG Ab	<9 SGU	<=20
Beta-2-Glycoprotein I IgM Ab	<9 SMU	<=20
Beta-2-Glycoprotein I IgA Ab	>150 SAU	<=20

One week after the last initial follow-up, the patient continued to follow up with hematology every three months. Since his last hospital discharge, he denied any additional stroke events. He has been on lifelong anti-coagulation therapy with rivaroxaban 20 mg once daily and clopidogrel 75 mg daily in combination with atorvastatin 40 mg daily.

## Discussion

In the clinical setting, the antiphospholipid antibodies (aPLs) detection tests included in antiphospholipid classification criteria are anticardiolipin (aCL) antibody (immunoglobulin G [IgG] or IgM) enzyme-linked immunosorbent assay (ELISA), anti-beta2-glycoprotein-I antibody (IgG or IgM) ELISA, and lupus anticoagulant (LA) assay. Anti-beta 2-glycoprotein-I (aβ2GPI) antibodies based on IgG and IgM isotypes are being given a particular focus in view of their recent inclusion in the antiphospholipid syndrome (APS) classification criteria [5].

There have been prospective studies focused on the estimation of IgG and IgM isotypes in patients with thromboembolic events, with only a few studies reporting on the significance of IgA isotype pathogenesis [6]. IgA isotypes are not particularly tested when evaluating for APS, and if they are reported, they are not considered as supportive evidence for APS diagnosis because they are not included in the consensus criteria [4]. It was concluded during the Laboratory Diagnostics and Trends APS Task Force of the 14th International Congress on aPL that there is a shred of low-quality evidence to include IgA isotype as part of the APS classification criteria, since these isotypes are usually associated with other aPLs, making it difficult to solely understand the role of IgA [3]. The efficacy of the IgA isotypes is generally restricted to those patients who have strong clinical suspicion for APS but who also tested negative for other tests for aPL [3].

On the contrary, the clinical importance of IgA anti-β2GPI antibodies has increased over the last 16 years [7]. One cohort study by Tortosa et al. followed up 244 patients who were positive for IgA-aβ2GPI without a history of APS-related symptoms for five years compared to 221 patients who tested negative for IgA-aβ2GPI. They found that patients who tested positive for IgA-aβ2GPI had a 13.8% risk of developing APS events compared to 3.2% risk of developing APS events in patients without IgA-aβ2GPI. Arterial thrombosis showed to be most frequently linked to APS-events (N=25, 55%) and was mainly observed in group-1 patients (21 vs. 4, p=0.001) [2]. Such a study has confirmed that the positive predictive value of IgA-aβ2GPI antibodies showed similar results to the value of IgG-aβ2GPI antibodies and higher than the positive value of IgM antibodies [4]. Thus, more prospective studies are needed to have a better understanding of the role of IgA anti-β2GPI as a thrombotic risk factor.

A study by Pierangeli et al. was conducted on a mouse model in vivo to study the role of antiphospholipid autoantibodies and immunoglobulins in the induction of thrombosis. Separate groups of mice were injected with affinity-purified IgG, IgM, and IgA anticardiolipin antibodies or with normal immunoglobulins of the same isotype, and the effects on thrombus formation were compared. The study showed that mean thrombus area and mean disappearance times were significantly increased in all four groups injected with affinity-purified antibodies [8].

It is evident that IgA anti-β2GPI antibodies have an association with many clinical manifestations of antiphospholipid syndrome and thrombotic events, particularly arterial thrombosis [9]. A study by Murthy et al. found 31.8% of patients with associated isolated IgA anti-β2GPI positivity for arterial thrombosis. After variables such as age, obesity, pregnancy, oral contraceptives use, end-stage renal disease, use of hydroxychloroquine or non-steroidal anti-inflammatory drugs, and smoking were all adjusted, isolated IgA anti-β2GPI positivity was significantly associated with arterial thrombosis, and strength of associated increased (Odds ratio of 5.8, adjusted p-value=0.003) [10].

The clinical significance of IgA isotypes of anti-β2GPI and aPL continues to be under investigation due to the lack of several prospective studies to evaluate the risk of isolated IgA-aβ2GPI on APS manifestations. These antibodies to date are not included in the classification criteria for APS [4]. However, in this clinical case discovered in early adulthood that showed the development of thrombotic events due to the presence of isolated IgA-aβ2GPI without a history of APS, and in support with few recent studies, it is possible to demonstrate that the presence of isolated IgA-aβ2GPI in asymptomatic patients seems to be a major risk factor in the development of APS events.

## Conclusions

IgA-aβ2GPI antibody is a rare phenomenon to induce recurrence of thrombogenesis, specifically in healthy young adults. Evaluating for APS-associated clinical manifestations due to IgA-aβ2GPI antibodies is often underestimated and unrecognized. The inclusion of these antibodies in aPL screening can explicitly further identify a group of patients with APS-associated clinical events, and such antibodies should be considered as a laboratory criterion for APS diagnosis. Thus, to determine the link between IgA-aβ2GPI antibodies and APS-events in asymptomatic individuals before recommending preventive treatment with anticoagulant or antiplatelet medications, there needs to be a broader intention to standardize IgA-aβ2GPI assays as a diagnostic criterion for APS.
